# The prognostic values of monitoring changes in coagulative, inflammatory, and blood chemistry markers in COVID-19 patient’s before and during admission to ICU: a retrospective cohort study

**DOI:** 10.1097/MS9.0000000000003600

**Published:** 2025-07-22

**Authors:** Amer Hashim Al Ani, Gabriel Andrade, Yara Elsherbiny, Afiya Walid Zaynob, Mesk Alhammadi, Kowthar Forsat, Vidya Jakapure

**Affiliations:** a Clinical and Medical Education, College of Medicine. Ajman University, Ajman, United Arab Emirates; b Sheikh Khalifa Medical City, Ajman, United Arab Emirates

**Keywords:** blood chemistry markers, coagulative, COVID-19, inflammatory, prognosis

## Abstract

**Introduction:**

The infection caused by the COVID-19 virus is associated with thromboembolic events and severe inflammatory reactions, significantly impacting the prognosis of infected patients. Numerous studies have indicated that COVID-19 patients often exhibit a hypercoagulable state, disseminated intravascular coagulation, and overwhelming inflammation, particularly in critically ill patients with multiple comorbidities requiring admission to the ICU. This study aims to assess the prognostic significance of alterations in coagulation, inflammatory, and blood chemistry markers in COVID-19 patients both before and during admission to the ICU.

**Methods:**

Study design and population: This retrospective observational cohort study was conducted from March 2020 to July 2021 at a single center, including 90 adult patients with confirmed COVID-19 infection requiring ICU admission. Patients were divided into two groups: survivors (*n* = 42) and non-survivors (*n* = 48). The median age of non-survivors was 48.5 years (BMI 26–40), while survivors had a median age of 54 years (BMI 23–35). All participants received uniform supportive therapy comprising endotracheal intubation, anticoagulation (low molecular weight heparin or unfractionated heparin), aspirin, and steroids. No antiviral therapy was administered. Inclusion criteria encompassed adult COVID-19-positive patients requiring ICU admission. Exclusion criteria included pediatric patients, adult COVID-19 patients not admitted to the ICU, and Intensive Care Unit (ICU) patients without COVID-19 infection. Data collection: Demographic data (age, gender, comorbidities) and laboratory parameters (D-dimer, lactate dehydrogenase [LDH], procalcitonin, prothrombin time, platelet count, ferritin, C-reactive protein [CRP], glucose, and creatinine) were extracted from electronic medical records at three time points: ICU admission, shortly after treatment initiation, and at discharge or death. Statistical analysis: A total of 94 patients were initially assessed; three were excluded due to incomplete data, yielding a final cohort of 91 patients. Missing data for certain variables were imputed using the median of respective variables. Given the non-normal distribution of most laboratory markers, non-parametric statistical tests were applied. Paired Wilcoxon signed-rank tests were used to compare biomarker medians between admission and subsequent time points. Mann–Whitney *U* tests were employed to evaluate differences between survivors and non-survivors. All tests were two-tailed with a significance threshold set at *P* ≤ 0.05. Analyses were performed using Jamovi software.

**Results:**

Baseline characteristics: A total of 91 patients were included in the final analysis, comprising 42 survivors (36 males [83.7%], 6 females [16.3%]; median age 54 years [Interquartile Range (IQR): 49–59]; Body Mass Index (BMI) range 23–35) and 48 non-survivors (40 males [83.3%], 8 females [16.7%]; median age 48.5 years [IQR: 45–53]; BMI range 26–40). Overall, the cohort was predominantly male (83.5%) and had a wide range of body mass index. At ICU admission, survivors had slightly higher median platelet counts (257 vs 254 × 10^9^/L) and ferritin levels (1491 vs 1212 ng/mL), whereas non-survivors had higher median D-dimer (3.33 vs 2.28 mg/L), CRP (185 vs 131 mg/L), and procalcitonin (0.825 vs 0.51 ng/mL) levels. Creatinine, LDH, and glucose levels were similar between the groups at admission. Baseline demographic and clinical characteristics, along with initial laboratory values, are summarized in Table 1. Temporal changes in biomarker levels: Serial measurements revealed significant biomarker changes across the ICU stay. In the overall cohort, the Wilcoxon signed-rank test identified significant increases in platelet count (median 256 to 294 × 10^9^/L, *P* < 0.001) and procalcitonin levels (median 0.6–0.93 ng/mL, *P* = 0.016) shortly after treatment initiation (Table 2). From admission to discharge or death, significant increases were observed in prothrombin time (median 14.5–15.2 s, p<0.001), procalcitonin (median 0.6–1.01 ng/mL, *P* < 0.001), and creatinine (median 78–92 µmol/L, *P* < 0.001), whereas CRP (median 172.5–61.2 mg/L, *P* < 0.001) and LDH (median 581–472 U/L, *P* = 0.001) significantly decreased (Table 3). These temporal dynamics are visually summarized in Figure 1 (panels A–E), displaying median and mean values with 95% confidence intervals for each biomarker. Comparisons between survivors and non-survivors: Mann–Whitney *U* test comparisons (Table 4) revealed significant differences between survivors and non-survivors. At admission, survivors had significantly lower glucose levels (median 10.8 vs 8.2 mmol/L, *P* = 0.006). Shortly after treatment, survivors exhibited lower D-dimer (*P* = 0.013), prothrombin time (*P* = 0.022), ferritin (*P* = 0.022), CRP (*P* = 0.028), and LDH (*P* = 0.003) levels compared to non-survivors. At discharge or death, survivors demonstrated significantly higher platelet counts (median 331 vs 211 × 10^9^/L, *P* < 0.001) and significantly lower D-dimer, prothrombin time, ferritin, CRP, procalcitonin, creatinine, and LDH levels (all *P* < 0.001). Subgroup analyses: Among non-survivors, significant increases in prothrombin time, ferritin, procalcitonin, and creatinine levels were observed between admission and shortly before death, alongside a decrease in platelet count (all *P* < 0.001). Conversely, survivors showed significant reductions in CRP, ferritin, procalcitonin, and glucose at discharge (all *P* < 0.001), accompanied by increased platelet counts (median 257–331 × 10^9^/L, *P* < 0.001) and decreased LDH (median 570–472 U/L, *P* = 0.001).

**Conclusion:**

This study identifies key biomarkers that predict COVID-19 outcomes, emphasizing the association between platelet count and the final fate of COVID-19 patients admitted to the ICU. Elevated ferritin levels predict disease deterioration and poor prognosis, whereas lower glucose levels indicate a better prognosis.

## Introduction

COVID-19 is associated with a spectrum of thromboembolic events, including venous thromboembolism^[1]^ and pulmonary embolism. Studies have indicated that severe COVID-19 is linked to heightened coagulation markers, potentially leading to disseminated intravascular coagulation and a cytokine storm^[2]^. Combining antithrombotic therapy such as Heparin or enoxaparin with antiviral treatment for COVID-19 patients is recommended, as research suggests it can reduce mortality rates by up to 30%^[3]^. Protocols advocate for the addition of anti-platelets like aspirin to help prevent thromboembolic events in COVID-19 patients in the ICU^[4]^, while also minimizing COVID-19 severity by reducing inflammatory markers and limiting platelet activation^[4]^. However, a major documented complication of these therapies is bleeding^[5]^. COVID-19 cases with comorbidities like renal failure are often treated with low molecular weight heparin^[6]^.

In healthy individuals, serum procalcitonin levels are typically undetectable by standard assays^[7]^. Observational studies have observed elevated levels of procalcitonin in patients with severe COVID-19 infection, though it remains unclear whether this elevation is related to secondary bacterial infection, the severity of the viral infection, or both^[7]^. Procalcitonin synthesis is substantially upregulated in multiple tissues in the presence of bacterial endotoxin or certain cytokines, including interleukin 6, contrasting with its response to viral infection^[7]^. Elevated procalcitonin in COVID-19 may signify bacterial co-infection, severity of acute respiratory distress syndrome, or increased cytokine production due to immune dysregulation from COVID-19–associated respiratory failure^[7]^.

Ferritin is known to accompany various acute infections, viral and bacterial alike, suggesting an acute response to inflammation^[8]^. Several retrospective studies have indicated that ferritin levels may correlate with and predict poor outcomes in COVID-19^[9]^. Patients with moderate and severe disease typically demonstrate a significant increase in ferritin levels compared to those with mild disease^[10]^. Given its potential to enhance the inflammatory process, ferritin could be explored as a novel therapeutic target to improve patient outcomes^[11]^.

C-reactive protein (CRP) levels tend to rise in COVID-19 patients due to inflammatory cytokines^[12]^. The use of low molecular weight heparin has been shown to reduce CRP levels owing to its anti-inflammatory effects^[12]^. Lactic dehydrogenase (LDH), present in lung tissue, is expected to be elevated in individuals with severe COVID-19 infections^[13]^. Activation of inflammasomes by SARS-CoV-2 leads to cellular pyroptosis and aggressive symptoms, partly explaining the association of LDH with COVID-19 patients^[14]^. Studies have demonstrated that medications like enoxaparin or fondaparinux can reduce LDH levels in COVID-19 patients^[15]^.

**HIGHLIGHTS**

- Changes in platelet count, ferritin, procalcitonin, and C-reactive protein (CRP) levels strongly correlate with COVID-19 patient survival in ICU.
- COVID-19 non-survivors showed decreasing platelet counts and persistently elevated ferritin, indicating disease progression.
- Elevated procalcitonin levels were consistently associated with mortality, supporting its role as a prognostic marker.
- COVID-19 Survivors exhibited significant reductions in CRP, ferritin, and lactic dehydrogenase during ICU stay.
- Despite standardized supportive therapy, outcomes varied significantly based on biomarker dynamics.

Meta-analyses have shown an association between COVID-19 and higher blood glucose levels^[16]^. Preexisting type 2 diabetic patients infected with the novel virus often require more medical interventions compared to non-diabetics, underscoring the importance of regular blood glucose monitoring to improve prognosis^[16]^. Poorly controlled hyperglycemia can significantly impact patient prognosis, as well as the severity and mortality of the infectious disease^[17]^.

This cohort study has been reported in line with the STROCSS guidelines^[18]^.

### Objectives

This study aims to assess the prognostic significance of monitoring changes in coagulation, inflammatory, and blood chemistry markers in COVID-19 patients both before and during admission to the ICU, as well as at the endpoint of treatment (recovery or mortality).

## Patients and methods

### Methods

#### Study design and population

This retrospective observational cohort study was conducted from March 2020 to July 2021 at a single center, including 90 adult patients with confirmed COVID-19 infection requiring ICU admission. Patients were divided into two groups: survivors (*n* = 42) and non-survivors (*n* = 48). The median age of non-survivors was 48.5 years (BMI 26–40), while survivors had a median age of 54 years (BMI 23–35). All participants received uniform supportive therapy comprising endotracheal intubation, anticoagulation (low molecular weight heparin or unfractionated heparin), aspirin, and steroids. No antiviral therapy was administered.

Inclusion criteria encompassed adult COVID-19-positive patients requiring ICU admission. Exclusion criteria included pediatric patients, adult COVID-19 patients not admitted to the ICU, and ICU patients without COVID-19 infection.

#### Data collection

Demographic data (age, gender, comorbidities) and laboratory parameters (D-dimer, lactate dehydrogenase [LDH], procalcitonin, prothrombin time, platelet count, ferritin, CRP, glucose, and creatinine) were extracted from electronic medical records at three time points: ICU admission, shortly after treatment initiation, and at discharge or death.

#### Statistical analysis

A total of 94 patients were initially assessed; three were excluded due to incomplete data, yielding a final cohort of 91 patients. Missing data for certain variables were imputed using the median of respective variables. Given the non-normal distribution of most laboratory markers, non-parametric statistical tests were applied. Paired Wilcoxon signed-rank tests were used to compare biomarker medians between admission and subsequent time points. Mann–Whitney *U* tests were employed to evaluate differences between survivors and non-survivors. All tests were two-tailed with a significance threshold set at *P* ≤ 0.05. Analyses were performed using Jamovi software.

This cohort study has been reported in line with the STROCSS guidelines^[18]^.

## Results

### Baseline characteristics

A total of 91 patients were included in the final analysis, comprising 42 survivors (36 males [83.7%], 6 females [16.3%]; median age 54 years [IQR: 49–59]; BMI range 23–35) and 48 non-survivors (40 males [83.3%], 8 females [16.7%]; median age 48.5 years [IQR: 45–53]; BMI range 26–40). Overall, the cohort was predominantly male (83.5%) and had a wide range of body mass index. At ICU admission, survivors had slightly higher median platelet counts (257 vs 254 × 10^9^/L) and ferritin levels (1491 vs 1212 ng/mL), whereas non-survivors had higher median D-dimer (3.33 vs 2.28 mg/L), CRP (185 vs 131 mg/L), and procalcitonin (0.825 vs 0.51 ng/mL) levels. Creatinine, LDH, and glucose levels were similar between the groups at admission. Baseline demographic and clinical characteristics, along with initial laboratory values, are summarized in Table 1.

**Table 1:** Median laboratory values of COVID-19 patients at admission, after treatment initiation, and at discharge or shortly before death, stratified by survival outcome

Biomarker	Entire sample					
	Admission		Post-treatment		Discharge/death	
D-dimer (mg/L)	2.87		5.06		3.76	
Prothrombin time (s)	14.5		14.9		15.2	
Platelet count (×10^9^/L)	256		294		266	
Ferritin (ng/mL)	1232		1172		1350	
CRP (mg/L)	173		121		61.2	
Procalcitonin (ng/mL)	0.6		0.93		1.01	
Glucose (mmol/L)	9.8		9.0		8.9	
Creatinine (µmol/L)	78		74		92	
LDH (U/L)	581		581		472	
**Biomarker**	**Survivors**			**Non-survivors**		
	**Admission**	**Post-treatment**	**Discharge**	**Admission**	**Post-treatment**	**Death**
D-dimer (mg/L)	2.28	3.58	3.31	3.33	6.92	5.84
Prothrombin time (s)	14.1	14.6	14.3	14.8	15.4	17.5
Platelet count (×10^9^/L)	257	294	331	254	299	211
Ferritin (ng/mL)	1491	1056	907	1212	1430	1881
CRP (mg/L)	131	85.1	25.1	185	171	112
Procalcitonin (ng/mL)	0.51	0.93	0.16	0.825	0.87	6.67
Glucose (mmol/L)	10.8	9.0	8.4	8.2	9.45	9.3
Creatinine (µmol/L)	77	68	67	78	85	153
LDH (U/L)	570	570	472	594	644	499

Data are presented as medians.

CRP: C-reactive protein; LDH, lactate dehydrogenase.

### Temporal changes in biomarker levels

Serial measurements revealed significant biomarker changes across the ICU stay. In the overall cohort, the Wilcoxon signed-rank test identified significant increases in platelet count (median 256–294 × 10^9^/L, *P* < 0.001) and procalcitonin levels (median 0.6–0.93 ng/mL, *P* = 0.016) shortly after treatment initiation (Table 2).

**Table 2:** Wilcoxon signed-rank test for changes in biomarkers after treatment initiation

Biomarker	Wilcoxon statistic	*P*-value
D-dimer (mg/L)	1610	0.342
Prothrombin time (s)	1324	**0.008**
Platelet count (×10^9^/L)	1124	**<0.001**
Ferritin (ng/mL)	2133	0.732
C-reactive protein (CRP, mg/L)	2576	0.056
Procalcitonin (ng/mL)	1448	**0.016**
Glucose (mmol/L)	2380	0.080
Creatinine (µmol/L)	1641	0.188
Lactate dehydrogenase (LDH, U/L)	1417	0.670

Wilcoxon signed-rank test statistics and *P*-values for changes in laboratory biomarkers from ICU admission to shortly after treatment initiation. Statistically significant differences are indicated in bold (*P* ≤ 0.05).

From admission to discharge or death, significant increases were observed in prothrombin time (median 14.5–15.2 s, *P* < 0.001), procalcitonin (median 0.6–1.01 ng/mL, *P* < 0.001), and creatinine (median 78–92 µmol/L, *P* < 0.001), whereas CRP (median 172.5-61.2 mg/L, *P* < 0.001) and LDH (median 581–472 U/L, *P* = 0.001) significantly decreased (Table 3).

**Table 3:** Wilcoxon signed-rank test for changes in biomarkers from admission to discharge or death

Biomarker	Wilcoxon statistic	*P*-value
D-dimer (mg/L)	1810	0.661
Prothrombin time (s)	748	**<0.001**
Platelet count (×10^9^/L)	1933	0.528
Ferritin (ng/mL)	1631	0.094
C-reactive protein (CRP, mg/L)	3182	**<0.001**
Procalcitonin (ng/mL)	1486	**0.016**
Glucose (mmol/L)	2220	0.277
Creatinine (µmol/L)	1475	**0.015**
Lactate dehydrogenase (LDH, U/L)	2283	**0.007**

Wilcoxon signed-rank test statistics and *P*-values comparing biomarker medians at ICU admission and at discharge or shortly before death. Statistically significant differences are shown in bold (*P* ≤ 0.05).

CRP, C-reactive protein; LDH, lactate dehydrogenase.

**Figure 1.**
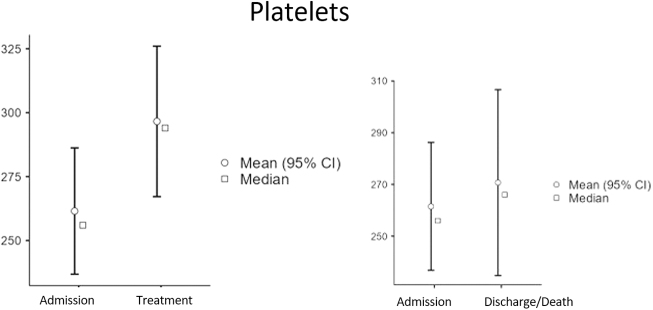
Temporal changes in biomarker levels in COVID-19 ICU patients. Changes in median (square symbols) and mean (circles, with 95% confidence intervals) values for key biomarkers are shown across three time-points: at ICU admission, shortly after treatment initiation, and at discharge or shortly before death. Platelet count, Biomarker levels in survivors and non-survivors are displayed to illustrate differential trajectories over the course of ICU stay.

These temporal dynamics are visually summarized in Figure 1 (panels A–E), displaying median and mean values with 95% confidence intervals for each biomarker.

### Comparisons between survivors and non-survivors

Mann–Whitney *U* test comparisons (Table 4) revealed significant differences between survivors and non-survivors.

**Table 4:** Mann–Whitney *U* test comparisons between survivors and non-survivors

	Admission	Post-treatment	Discharge/death
Biomarker (unit)	U statistic (*P*-value)		
Age (years)	**782 (0.046)**	–	–
D-dimer (mg/L)	935 (0.433)	**721 (0.013)**	**588 (<0.001)**
Prothrombin time (s)	811 (0.076)	**745 (0.022)**	**291 (<0.001)**
Platelet count (×10^9^/L)	878 (0.217)	960 (0.559)	**610 (<0.001)**
Ferritin (ng/mL)	935 (0.432)	**746 (0.022)**	**424 (<0.001)**
CRP (mg/L)	801 (0.065)	**756 (0.028)**	**366 (<0.001)**
Procalcitonin (ng/mL)	811 (0.077)	912 (0.335)	**204 (<0.001)**
Glucose (mmol/L)	**685 (0.006)**	941 (0.460)	965 (0.584)
Creatinine (µmol/L)	1026 (0.949)	790 (0.053)	**466 (<0.001)**
LDH (U/L)	891 (0.256)	**660 (0.003)**	**629 (0.001)**

Mann–Whitney *U* test comparisons between survivors and non-survivors at three key time-points: admission, shortly after treatment initiation, and at discharge or shortly before death. Data are presented as the Mann–Whitney *U* test statistic followed by the *P*-value in parentheses (*U* [*P*-value]). Statistically significant differences (*P* ≤ 0.05) are highlighted in bold.

CRP, C-reactive protein; LDH, lactate dehydrogenase.

At admission, survivors had significantly lower glucose levels (median 10.8 vs 8.2 mmol/L, *P* = 0.006). Shortly after treatment, survivors exhibited lower D-dimer (*P* = 0.013), prothrombin time (*P* = 0.022), ferritin (*P* = 0.022), CRP (*P* = 0.028), and LDH (*P* = 0.003) levels compared to non-survivors. At discharge or death, survivors demonstrated significantly higher platelet counts (median 331 vs 211 × 10^9^/L, *P* < 0.001) and significantly lower D-dimer, prothrombin time, ferritin, CRP, procalcitonin, creatinine, and LDH levels (all *P* < 0.001).

### Subgroup analyses

Among non-survivors, significant increases in prothrombin time, ferritin, procalcitonin, and creatinine levels were observed between admission and shortly before death, alongside a decrease in platelet count (all *P* < 0.001). Conversely, survivors showed significant reductions in CRP, ferritin, procalcitonin, and glucose at discharge (all *P* < 0.001), accompanied by increased platelet counts (median 257–331 × 10^9^/L, *P* < 0.001) and decreased LDH (median 570–472 U/L, *P* = 0.001).

## Discussion

The seventh human coronavirus (SARS-CoV-2) has resulted in a significant number of fatalities worldwide^[19]^. Despite extensive research on this pathogen, there remains a dearth of literature discussing its laboratory and prognostic implications^[20]^. Many studies have faced challenges in drawing conclusive findings on the prognostic laboratory values of COVID-19 due to limitations in their study designs. In this investigation, our objective was to assess the prognostic significance of specific laboratory parameters in ICU-admitted patients infected with COVID-19, and to identify any correlations between these parameters and patient survival.

**Figure 2.**
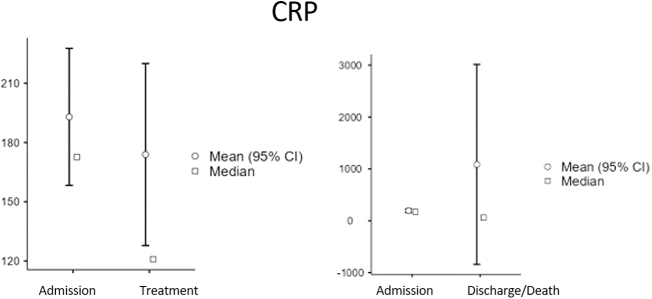
Temporal changes in biomarker levels in COVID-19 ICU patients. Changes in median (square symbols) and mean (circles, with 95% confidence intervals) values for key biomarkers are shown across three time-points: at ICU admission, shortly after treatment initiation, and at discharge or shortly before death. CCRP levels in survivors and non-survivors are displayed to illustrate differential trajectories over the course of ICU stay.

Our study reveals that elevated levels of inflammatory markers (CRP and LDH) (Fig. 2) and a reduction in platelet count are correlated with increased severity of COVID-19. These findings align with a meta-analysis comprising 148 studies, which reported similar results^[21]^. Inflammatory markers are established predictive indicators of disease severity and exhibit a strong association with COVID-19 prognosis. A decrease in CRP levels signifies a favorable prognosis and often leads to the discharge of patients from the ICU.

Regarding coagulopathy, viruses are known to influence platelet count and size^[22]^. Our study demonstrates a significant disparity in platelet levels between patients who succumbed to the illness and those who were discharged from the ICU. Higher platelet levels are associated with patient discharge, whereas lower levels correlate with mortality (Fig. 1). Reduced platelet levels indicate heightened platelet activation and thrombus formation^[23]^. A cohort study involving 3915 hospitalized COVID-19 patients yielded consistent findings with our investigation^[24]^, highlighting the association of platelet activity markers with severe outcomes.

**Figure 3.**
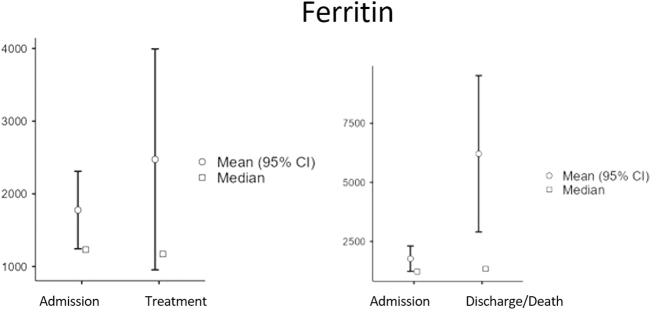
Temporal changes in biomarker levels in COVID-19 ICU patients. Changes in median (square symbols) and mean (circles, with 95% confidence intervals) values for key biomarkers are shown across three time-points: at ICU admission, shortly after treatment initiation, and at discharge or shortly before death. Ferritin and otherbiomarker levels in survivors and non-survivors are displayed to illustrate differential trajectories over the course of ICU stay.

Procalcitonin has emerged as a valuable prognostic biomarker for COVID-19^[25]^. Our study reaffirms a clear association: elevated procalcitonin levels indicate a poor prognosis and are linked to patient mortality, whereas lower levels are associated with patient discharge (Fig. 3). This concurs with several studies indicating that elevated procalcitonin values are indicative of severe COVID-19^[25–29]^. According to a meta-analysis, a significant rise in serum procalcitonin levels suggests bacterial co-infection, leading to a more severe disease course and complex clinical presentation^[30]^.

**Figure 4.**
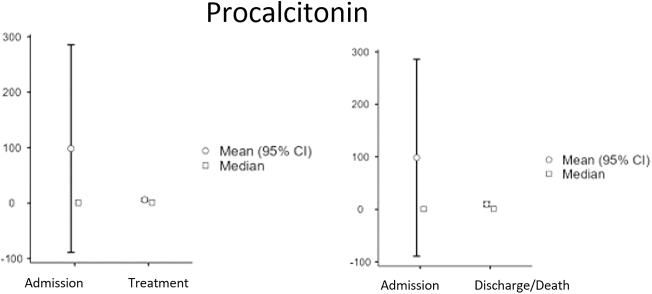
Temporal changes in biomarker levels in COVID-19 ICU patients. Changes in median (square symbols) and mean (circles, with 95% confidence intervals) values for key biomarkers are shown across three time-points: at ICU admission, shortly after treatment initiation, and at discharge or shortly before death. Procalcitonin levels in survivors and non-survivors are displayed to illustrate differential trajectories over the course of ICU stay.

Ferritin serves as a key regulator of the immune system and directly contributes to cytokine storm development^[31]^. In our investigation, both groups of patients (those discharged and those deceased) exhibited elevated ferritin levels from admission until the initiation of treatment. However, discharged patients demonstrated a decline in serum ferritin levels, whereas deceased patients showed elevated levels (Fig. 4). This underscores the pivotal role of serum ferritin in influencing COVID-19 severity. Similar findings are documented in a meta-analysis involving 10 614 patients, wherein elevated serum ferritin levels were identified as predictors of worsening COVID-19 and poor prognosis^[32]^.

**Figure 5.**
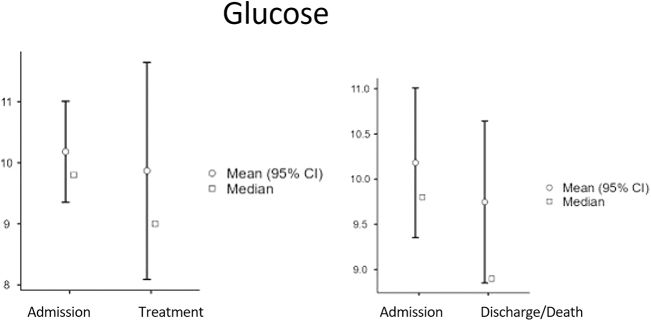
Temporal changes in biomarker levels in COVID-19 ICU patients. Changes in median (square symbols) and mean (circles, with 95% confidence intervals) values for key biomarkers are shown across three time-points: at ICU admission, shortly after treatment initiation, and at discharge or shortly before death. Glucose levels in survivors and non-survivors are displayed to illustrate differential trajectories over the course of ICU stay.

Glucose levels often serve as independent predictors of COVID-19 mortality and morbidity^[17]^. Our study did not reveal any significant association between elevated glucose levels and poor prognosis. Conversely, low blood glucose levels were indicative of a better prognosis (Fig. 5). A meta-analysis corroborated these results, demonstrating that well-controlled blood glucose levels improve patient outcomes^[33]^. Another study highlighted the significant impact of poorly controlled blood glucose levels on increasing COVID-19 severity and mortality^[17]^, findings not observed in our study.

## Conclusion

Understanding the prognostic laboratory markers in COVID-19-infected patients is of paramount importance. Close monitoring can facilitate early diagnosis and treatment, thereby averting the progression to a more severe form of the disease and improving outcomes. Our findings indicate that most survivors of COVID-19 admitted to the ICU exhibited lower levels of platelets, ferritin, procalcitonin, glucose, and creatinine, in contrast to non-survivors who demonstrated higher levels of these markers. Further research will enhance our understanding of these laboratory prognostic indicators in COVID-19-infected ICU patients.

### Limitations of the study

This study was conducted on a relatively small number of patients and does not constitute a systematic review to definitively establish the impact of these markers on survival.

## Data Availability

I confirm that the datasets generated during and/or analyzed during the current study are publicly available, available upon reasonable request.

## Research registration unique identifying number (UIN)

Assigned unique identifying number (UIN): Research registry: 9423 on August 2023.
